# PB.35. How does semi-automated computer-derived CT measure of breast density compare with subjective assessments to measure mean glandular breast density in patients with breast cancer?

**DOI:** 10.1186/bcr3714

**Published:** 2014-11-03

**Authors:** G Bansal, S Yapa

**Affiliations:** 1 University Hospital Llandough, Penarth, UK

## Introduction

The objectives were to compare radiologists' breast mammographic density readings with computed tomography (CT) subjective measures; and to correlate computer-derived measurement of CT density with subjective assessments.

## Methods

Retrospective review of mammograms and CT scans in 77 breast cancer patients obtained within 1 year of each other was performed. Two radiologists independently reviewed both CT and mammograms and classified each case into four categories as defined by the Breast Imaging, Reporting and Data system of the American College of Radiology. Inter-reader agreements were obtained for both mammographic and CT density subjective evaluations by using the Cohen weighted *k *statistic. Correlation was also sought between subjective CT density measurement and mammographic density measurements for each reader using Spearman correlation coefficient. The semi-automated computer-derived measurement of breast density was correlated with visual measurements.

## Results

Inter-reader agreements was lower for subjective CT density grades than for mammographic readings, 0.428 (CI: 0.24 to 0.89) versus 0.571 (CI 0.35 to 0.76), respectively. There was moderately good correlation between subjective CT density grades and the mammographic density grades for both readers (0.760, Reader 1; 0.913, Reader 2). The semi-automated CT density measurement correlated well with the subjective assessments, with complete agreement of the density grades in 84.9% of patients and only one-level difference in the rest.

## Conclusions

Semi-automated CT density measurements in the evaluation of breast density correlated well with subjective mammographic density measurement. Further studies are needed to incorporate this extra information from a CT scan in the risk stratification of patients.

